# Red Blood Cell Contribution to Thrombosis in Polycythemia Vera and Essential Thrombocythemia

**DOI:** 10.3390/ijms25031417

**Published:** 2024-01-24

**Authors:** Julien M. P. Grenier, Wassim El Nemer, Maria De Grandis

**Affiliations:** 1 Etablissement Français du Sang PACA-Corse, Aix Marseille University, CNRS, ADES UMR 7268, 13005 Marseille, France; 2 Laboratoire d’Excellence GR-Ex, 75015 Paris, France

**Keywords:** red blood cell, polycythemia vera, essential thrombocythemia, thrombosis, adhesion

## Abstract

Polycythemia vera (PV) and essential thrombocythemia (ET) are myeloproliferative neoplasms (MPN) characterized by clonal erythrocytosis and thrombocytosis, respectively. The main goal of therapy in PV and ET is to prevent thrombohemorrhagic complications. Despite a debated notion that red blood cells (RBCs) play a passive and minor role in thrombosis, there has been increasing evidence over the past decades that RBCs may play a biological and clinical role in PV and ET pathophysiology. This review summarizes the main mechanisms that suggest the involvement of PV and ET RBCs in thrombosis, including quantitative and qualitative RBC abnormalities reported in these pathologies. Among these abnormalities, we discuss increased RBC counts and hematocrit, that modulate blood rheology by increasing viscosity, as well as qualitative changes, such as deformability, aggregation, expression of adhesion proteins and phosphatidylserine and release of extracellular microvesicles. While the direct relationship between a high red cell count and thrombosis is well-known, the intrinsic defects of RBCs from PV and ET patients are new contributors that need to be investigated in depth in order to elucidate their role and pave the way for new therapeutical strategies.

## 1. Introduction

Philadelphia chromosome-negative myeloproliferative neoplasms (MPNs) are a group of blood disorders characterized by clonal expansion of abnormal hematopoietic stem/progenitor cells resulting in an overabundance of erythrocytes, white blood cells (WBCs) and platelets. The two most common MPNs are polycythemia vera (PV), in which patients present increased red cell mass, often associated with increased platelet and white cell counts, and essential thrombocythemia (ET), which is defined by an elevated platelet count but normal red cell mass [1]. The dysregulation of the JAK/STAT pathway is the mechanistic hallmark of MPNs and is caused by somatic mutations in driver genes including JAK2, CALR and MPL. In 95% of patients with PV and 60% of those with ET, a single somatically acquired mutation is found in the gene encoding JAK2 [2,3,4,5], resulting in the V617F substitution at the protein level that dysregulates the kinase activity and drives the ligand-independent activation of receptor signaling. In JAK2V617F-negative PV cases, somatic gain-of-function mutations were found in JAK2 exon 12 conferring EPO-independent autonomous growth and EPO-hypersensitivity to bone marrow colonies, both in vitro and in vivo [6]. Compared to the V617F mutation, the exon 12 mutation results in stronger ligand-independent signaling and patients are characterized by higher hemoglobin levels and isolated erythrocytosis [7].

The major causes of morbidity and mortality in PV and ET are arterial and venous complications, progression to myelofibrosis and transformation to acute leukemia.

The pathogenesis of thrombosis results from a complex interplay of clinical and disease-related factors. Abnormalities of blood cells arising from the clonal proliferation of mutated hematopoietic stem cells involve not only quantitative changes but also qualitative modifications that characterize the switch of these cells from a resting to a procoagulant phenotype. Several studies indicate that vascular risk is substantially increased in PV and ET patients, even in relatively young patients with no previous vascular history, and becomes very high in older patients with a prior vascular event. The incidence range of arterial and venous thrombosis is 12–39% in PV and 11–25% in ET [8], with continuous efforts to disclose the underlying mechanisms. In many instances, thrombosis first occurs at the time of diagnosis, but cardiovascular events continue to occur during follow-up, even in treated patients. Thromboses are both arterial and venous and may occur virtually in any area. Arterial thrombosis, including acute myocardial infarction, cerebrovascular ischemic episodes and peripheral arterial occlusion, represent 60 to 70% of all cardiovascular events in PV. PV patients also experience deep vein thrombosis and pulmonary embolism. Moreover, they show a high prevalence of rare forms of thrombosis, such as abdominal vein thrombosis, including extrahepatic portal vein occlusion, Budd–Chiari syndrome and mesenteric vein thrombosis [9].

Risk stratification in PV and ET has been designed to estimate the likelihood of thrombotic complications [10]. Predictors of arterial complications include age > 60 years, leukocytosis >11 G/L, prior history of thrombosis, and cardiovascular risk factors [11]. High-risk PV and ET patients require cytoreductive therapy, while low-risk patients require daily aspirin therapy [10]. The first-line drug of choice for cytoreductive therapy, in both PV and ET, is hydroxycarbamide (HC), while the second-line drugs of choice include pegylated-interferon alpha (IFN) [10].

The pathophysiology of thrombosis in PV and ET is multifactorial and complex, a variety of blood cells have been reported to participate in this mechanism. In PV, platelets and leukocytes show a number of morphological, functional and biochemical abnormalities. PV platelets have increased surface expression of P-selectin, thrombospondin and activated fibrinogen receptor GPIIb/IIIa [12,13]. Moreover, they show an abnormal activation associated with modified cell surface localization and the stability of the cMPL receptor [14]. As a matter of fact, it has been shown that JAK2 mutations impair cMPL signal transduction for TPO-induced platelet priming leading to chronic platelet hyperresponsiveness [15]. Increased WBC count is associated with vascular risk in PV and ET patients [16]. In addition to the cell count, abnormal leukocyte activation is found in these patients. Activated leukocytes release an array of substances as reactive oxygen species (ROS) and proteolytic enzymes contributing to the activation of the hemostatic system, the inactivation of physiological inhibitors of coagulation and the formation of neutrophil/platelet complexes [17]. Platelet and leukocyte activation perturb the resting state of the endothelium and turns it into a prothrombotic surface. This damage triggers the release of Von Willebrand factor (vWF) in the circulation which in turn binds to platelets and activates their aggregation and the subsequent reinforcement of the clot in an amplification loop [18]. The presence of the JAK2V617F mutation was reported in endothelial cells (ECs) [19], with in vivo and in vitro studies showing the pro-thrombotic nature of the cells bearing the mutation [20,21]. In these studies, mice with endothelial-specific JAK2V617F expression displayed a higher propensity for thrombus formation due to the pro-adhesive phenotype of mutated ECs, associated with the increased expression levels of vWF and P-selectin (CD62P) [20,21].

For a long time, red blood cells (RBCs) were considered to be passive contributors to thrombotic events [22]. In recent years, clinical and epidemiological studies have associated quantitative and qualitative abnormalities of RBCs with both arterial and venous thrombosis. A growing body of mechanistic studies suggests that RBCs can promote thrombus formation and enhance thrombus stability. These findings suggest that RBCs may contribute to the pathophysiology of thrombosis, paving the way to potential therapeutical strategies targeting RBCs to prevent thrombosis or reduce its incidence [23]. In the context of MPNs, several RBC-related abnormalities have been reported, such as increased RBC count, altered RBC deformability, abnormal expression and activation of adhesion proteins, and release of extracellular microvesicles. These abnormalities contribute to increased vascular resistance and enhanced interaction of RBCs with the vascular wall, which may contribute to thrombus formation. In this review, we summarize the main findings of the literature that suggest an active role for RBCs in PV and ET thrombotic complications.

## 2. Quantitative and Qualitative Features of PV and ET RBCs in Relation to Thrombosis

RBCs are the most abundant cell type in the body. Their primary role is to transport oxygen to the tissues and carbon dioxide to the lungs. With their flexible structure, RBCs are capable of deforming in order to travel through all blood vessels including very small capillaries. Throughout the 120 days of their lifespan in circulation, RBCs travel in the bloodstream and come in direct or indirect contact with almost all of the cell types of the organism. For a long time, RBCs were considered to be passive carriers of respiratory gases; however, emerging evidence highlights a more complex physiological role. This was brought by recent discoveries demonstrating altered RBC functions in the context of hematological and non-hematological disorders.

### 2.1. Elevated Hematocrit and Rheological Parameters

**Figure 1:**
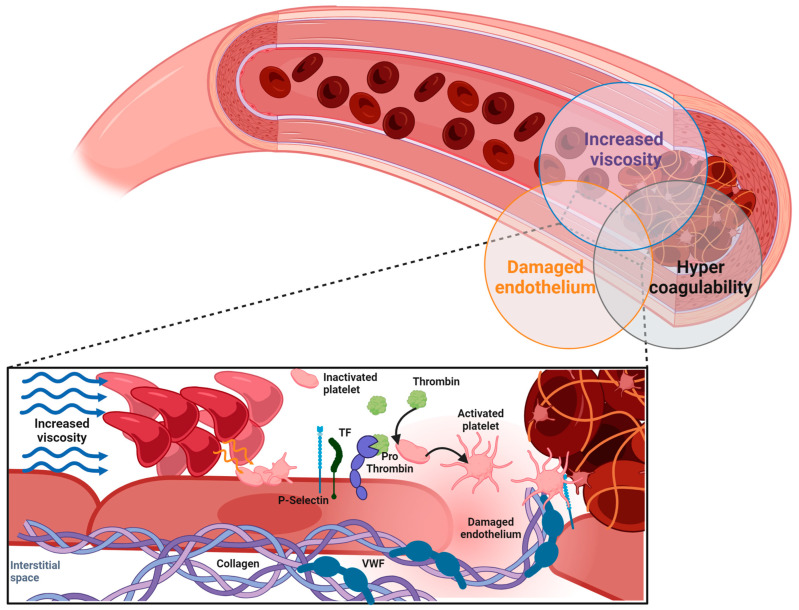
Thrombus generation: TF: tissue factor; VWF: Von Willebrand factor.

Being the major cellular component of blood, RBCs play a central role in defining and regulating the rheological parameters of the bloodstream. Hematocrit (HCT) represents the ratio of the volume of RBCs to the total volume of the blood [24]. Walton et al. showed that mice with high HCT, irrespective of platelet count and thrombin generation, had a reduced bleeding time after injury and a faster artery occlusion arising from accelerated platelet accumulation in the vicinity of the clot [25]. One of the major criteria in PV diagnosis is the presence of elevated HCT resulting from excessive erythrocytosis. The prothrombotic role of elevated HCT has been clearly demonstrated in PV [26,27], as patients maintaining an HCT target of less than 45% showed a lower rate of thrombotic complications as compared to those with a target of 45 to 50% [28]. Moreover, using a PV mouse model, it was shown that stalled blood flow in brain capillaries was correlated with high HCT [29]. Elevated HCT contributes to thrombotic complications by increasing blood viscosity, reducing blood return through the venous system and increasing platelet adhesion. Increased blood viscosity promotes blood clot formation and increases platelet activation at the vessel wall. Under low shear rates, as in the venous bed, hyperviscosity causes a major disturbance to the blood flow and at high shear rates, as in the arteries, the increase in the red cell mass displaces platelets toward the vessel wall, thus facilitating shear-induced platelet activation and enhancing platelet–platelet interactions. The pathogenesis of a thrombus is multifactorial, and it is admitted that its occurrence is mainly determined by the interrelation of the three physiological factors of Virchow’s triad: the hypercoagulability of the blood, the stasis of blood flow and the intravascular damage of the vessel wall. Erythrocytosis has a negative effect on all components of Virchow’s triad (Figure 1). The elevated RBC count in PV is responsible for increased viscosity, slowing down the blood flow, acting as a strong prothrombotic factor. All together, these observations suggest that preserving a normal hematocrit in these patients might further diminish the occurrence of serious thrombotic complications. However, thrombotic events could be still observed in some patients under cytoreductive treatment [30] suggesting that besides hematocrit, other parameters contribute to thrombosis physiopathology in PV.

### 2.2. Altered Deformability

The rheological properties of RBCs play an important role in their microcirculation. Deformability is an essential feature of RBCs that enables them to circulate through the smallest capillaries of the human body. Physiologically, RBCs, that have a 7–8 μm diameter, change their native biconcave shape to a bullet-like shape every time they squeeze through 1–3 μm blood vessels. This deformation is critical to ensure a high interface area with the vessel wall that is necessary for the efficient exchange of oxygen and carbon dioxide between the blood and tissues. Deformability is a function of (i) the structural proteins of the cytoskeleton, (ii) processes controlling intracellular ion and water handling and (iii) the membrane surface-to-volume ratio.

**Figure 2:**
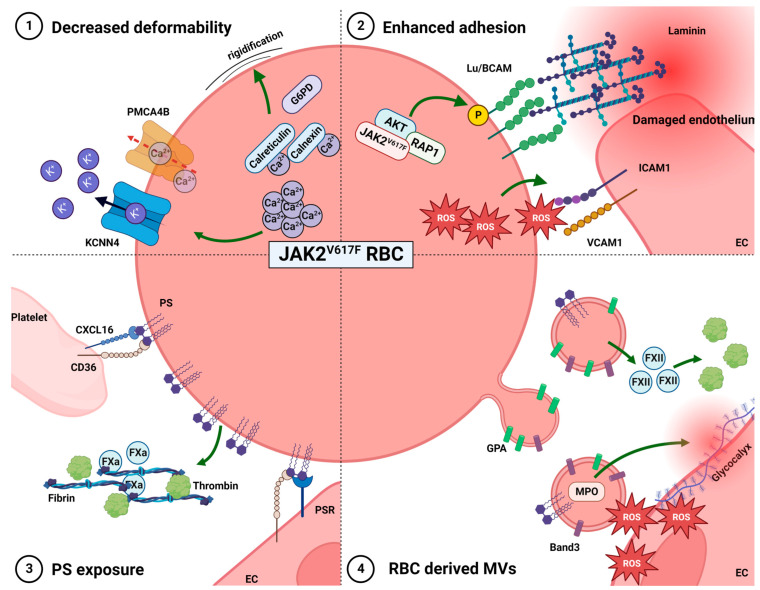
Contribution of JAK2 mutant RBCs to thrombus pathogenesis: EC: endothelial cells; PS: phosphatidyl serine; PSR: phosphatidyl serine receptor; MVs: microvesicles.

The deformability of RBCs is critical for the blood flow, even a slight decrease in RBC deformability may cause a significant increase in microvascular flow resistance and blood viscosity, which may lead to thrombotic complications. Investigations of PV RBC morphodynamics using laser-assisted approaches have shown a significant reduction in PV RBC deformability [31]. Dąbrowski and colleagues showed an elevated activity of glucose-6-phosphate dehydrogenase and acetylcholinesterase in PV RBCs that together with elevated levels of glutathione and malonyldialdehyde can explain their increased rigidity [32] (Figure 2). Ektacytometry experiments using LoRRca (laser-optical rotational red cell analyzer), confirmed the reduced deformability and elasticity of PV RBCs and showed increased aggregation amplitude compared to those from heathy donors [31]. Interestingly, this increased aggregation discriminates patients with a high risk of stroke among PV patients [33].

Intracellular Ca^2+^ is a potent effector of RBC biophysical and rheological properties that may play a role in thrombus formation [34]. Several studies have shown that calcium homeostasis may be altered in PV RBCs, with (i) higher levels of free intracellular Ca^2+^ as compared to healthy RBCs [35], (ii) elevated levels of Ca^2+^-binding proteins, such as calnexin and calreticulin and (iii) reduced levels of ATPase plasma membrane Ca^2+^ transporting 4 (PMCA4B), responsible for Ca^2+^ export [36]. Increased free intracellular Ca^2+^ levels in PV RBCs may lead to increased Gárdos channel activity, promoting potassium efflux and subsequent cell dehydration and rigidification (Figure 2). Intracellular Ca^2+^ accumulation in PV RBCs seems to be associated with the presence of JAK2V617F, which attributes a role for this mutation in the alteration of RBC biochemical and biomechanical properties, that in turn may contribute to thrombosis.

### 2.3. Enhanced RBC Adhesion to the Vascular Endothelium

In physiological conditions, interactions and adhesion events between circulating RBCs, endothelial cells and other circulating cells are minimal. In some pathological conditions, RBCs were reported to be sticky and to activate the cells of the vascular bed resulting in vaso-occlusion, as observed in sickle-cell disease [37,38,39,40]. Unexpectedly for a circulating non-adhesive cell, the RBC expresses a panel of adhesion molecules at its surface. These proteins play an important role in normal red cell development and some of them contribute to clinical complications in pathophysiology. Selectin receptors expressed on RBCs actively participate in leukocytes “rolling” on endothelial cells whereas intercellular adhesion molecule 4 (ICAM-4), CD44, and Lutheran/basal cell adhesion molecule (Lu/BCAM) are involved in firm interactions. It was shown that sickle RBCs could bind to the subendothelial matrix exposed after endothelial damage, partly through an interaction involving Lu/BCAM [41,42]. Of note, the phosphorylation of serine 621 in the cytoplasmic tail of Lu/BCAM is responsible for the activation of Lu-mediated cell adhesion to laminin 511/521 [43]. Wautier et al. showed increased adhesion of RBCs from PV patients to endothelial cells under static and flow conditions [44] (Figure 2). This increased adhesion was mediated by erythroid Lu/BCAM and endothelial laminin 511/521. Moreover, they showed that in PV RBCs Lu/BCAM was overexpressed and constitutively phosphorylated through a JAK2V617F/Rap1/Akt-signaling pathway targeting serine 621 [45] (Figure 2). In vivo, the shear stress in the postcapillary venules varies from 0.1 to 0.5 Pa; the adhesion of PV RBCs is established in this range and a proportion of adherent cells could withstand such forces suggesting the existence of a high-affinity adhesive interaction. In addition to Lu/BCAM, PV RBCs show the abnormal expression of several proteins, including proteins from the endoplasmic reticulum, such as calreticulin and calnexin, and transporters, like ATP binding cassette Subfamily G member 2 (ABCG2) [36], that could directly or indirectly affect cell surface properties. Moreover, elevated values of malonyldialdehyde associated with lipid peroxidation and reactive oxygen species (ROS) production were described in PV RBCs [32] (Figure 2). Such high values in a PV context may promote RBC adhesion to endothelial cells as ROS production is known to increase the expression of cell adhesion molecules intercellular adhesion molecule 1 (ICAM-1) and vascular cell adhesion molecule 1 (VCAM-1) on the vascular endothelium through NF-kB activation [46] (Figure 2). Pegylated-interferon alpha (IFN) and hydroxycarbamide (HC) are commonly used to treat PV and ET. HC efficacy in preventing thrombosis was suggested in several randomized clinical trials but is still not proven [28]. HC but not IFN enhances the expression of several proteins in PV red cell ghosts, including Lu/BCAM and CD147 adhesion proteins, and further exacerbates RBC adhesion to laminin in vitro [47].

### 2.4. Phosphatidylserine Exposure

Phosphatidylserine (PS) is an amino-phospholipid known to play a crucial role in mediating the recognition of senescent RBCs, serving as an eat-me signal. During aging, upon injury of cells or under certain pathological conditions, scramblase translocates PS from the inner to the outer leaflet, leading to increased concentrations on the external surface [48]. Peyrou et al. suggested that these PS-expressing RBCs may be a source of thrombin generation through the meizothrombin pathway and may play an active role in clot formation and stabilization [49] (Figure 2). PS-expressing RBCs could also contribute to thrombus formation via platelet activation. In anemic mice, a small population of RBCs was located in platelet thrombi and RBC/platelet interaction via FasL/FasR induced externalization of PS on the RBC membrane and enhanced platelet activation were observed [50].

PV patients are characterized by increased PS exposing RBCs/platelets, which could contribute to their hypercoagulable state [51]. While ~0.5–0.6% of the RBC population normally express PS in healthy subjects, the contribution of RBCs in pathological conditions may reach 40% of the thrombin-generating potential of whole blood [52]. Several studies using static adhesion assays or flow-based adhesion models have indicated that PS could participate to RBC adherence to endothelium [53,54,55]: RBCs can directly bind to CD36 and PS-receptor (PSR) on ECs [56] as well as to Chemokine (C-X-C motif) ligand 16 (CXCL16) or CD36 present on activated platelets [57] (Figure 2). The levels of PS-exposing RBCs are also higher in ET patients compared to healthy donors, with the highest levels observed in ET patients harboring JAK2 mutations. PS exposing RBCs were shown to contribute to the hypercoagulable state in ET patients by increasing the production of Factor Xa (FXa), thrombin and fibrin [58] (Figure 2).

### 2.5. RBC-Derived Microvesicles

Microvesicles (MVs) derived from RBCs are membranous extracellular structures shed into plasma with a diameter of 50–200 nm. They play an important role as key mediators of intercellular communication and consequently have an impact on various physiological processes such as blood homeostasis [59]. RBC-derived MVs expose on their surface antigens derived from the RBC membrane such as Glycophorin A (GPA) and Band 3, as well as PS (Figure 2). It was shown that RBC-derived MVs accumulate during blood storage and might be responsible for an increased incidence of deep vein thrombosis or other thrombotic conditions after transfusion of blood units that have been stored for a long duration [60]. Moreover, higher levels of MVs are associated with a dose- and time-dependent increase of thrombin generation and a reduction in clotting time, suggesting that they enhance hypercoagulability [61]. This enhanced thrombin generation has been associated with the expression of PS [62]. Alternatively, RBC-derived MVs can initiate thrombin formation either via a factor XII-dependent pathway or via the direct activation of FIX in a kallikrein-dependent manner [63] without tissue factor activity [64] (Figure 2). The circulating MVs can also promote vaso-occlusion by internalizing free heme and transferring it to the vascular endothelium or activating the complement system [65].

MPN patients have higher plasmatic levels of MVs than healthy individuals, with the MVs being derived from RBCs, platelets and endothelial cells. MV levels were shown to be higher in MPN patients having experienced thrombotic events [66,67]. In addition to this quantitative parameter related to their concentration in the circulation, MVs show a membrane and content composition that is specific to PV patients. As a matter of fact, JAK2V617F RBC-derived MVs were shown to be responsible for increased endothelial oxidative stress and nitric oxide (NO) pathway inhibition, increasing arterial contraction that accounts for the arterial events associated with the disease. At the molecular level, PV RBC-derived MVs have a defect in glutathione S-transferase theta 1 (GSTT1) and an overexpression of myeloperoxidase (MPO) [68] (Figure 2). MPO disrupts vascular endothelium by destabilizing the glycocalyx, resulting in neutrophil recruitment and the generation of neutrophil extracellular traps (NETs) [69]. NETs are directly involved in thrombus generation via various mechanisms that have been reviewed elsewhere [70]. A various number of clinical studies have shown that serum MPO and DNA levels are associated with an increased risk of deep vein thrombosis and pulmonary embolism in humans [71,72,73,74] suggesting that MPO levels might be used as a biomarker in patients with PV and ET.

A recent paper associates increased levels of RBC-derived MVs with pro-thrombotic microenvironment in patients with type 2 diabetes mellitus and suggests monitoring RBC-derived MVs levels by flow-cytometry as a biomarker of thrombotic risk [75]; the possibility needs to be further investigated in PVs and ETs.

### 2.6. Clinical Relevance of RBC Properties in Thrombotic Risk 

In MPNs, thrombotic risk correlates with age, prior thrombosis, hypertension, hyperlipidemia, leukocytosis, JAK2V617F allele burden and therapy in some, but not all, studies, but only age and prior thrombosis are included in thrombosis risk stratification. Recently, several studies reported that RBC distribution width (RDW) predicts thrombosis in patients with PV [76,77,78,79]. RDW is a parameter that measures the size and the volume of RBCs, but the relation between RDW values and thrombotic events remains unclear. However, it has been demonstrated that higher RDW values are associated with decreased RBC deformability [80] leading to erythrocytes aggregation, increased blood viscosity and thrombotic susceptibility [81,82].

## 3. Conclusions and Perspectives

The ability of RBCs to promote thrombosis is multifactorial, with several underlying mechanisms that are probably needed in concert. Although RBCs are not known to be the drivers of thrombotic events, the studies cited in this review are important elements supporting the active role of RBCs in this pathological manifestation in PV and ET. While the direct relationship between a high red cell count and thrombosis is well-known, the intrinsic defects of RBCs from PV and ET patients are new contributors that need to be investigated in depth in order to elucidate their role and pave the way for new biomarkers (Table 1) and therapeutical strategies.

## Figures and Tables

**Table 1:** RBC-related biomarkers for prediction of thrombotic events in PV and ET patients.

Parameter	Read Out	Clinical Routine Compatible
Hematocrit	Blood count	yes
RBCs deformability	LoRRca	yes
Ca^2+^ concentration	Fluorescence/Ca^2+^ chelator/ atomic absorption microscopy	difficult
Intracellular proteins	Western Blot/FACS	no
Adhesion molecules	FACS	difficult
PS exposure	FACS	yes
RBC derived MVs	FACS	yes
Serum MPO	ELISA	yes
Oxidative stress	qPCR/fluorescence	difficult
